# Stronger together: perspectives on gratitude social processes in group interventions for adolescents

**DOI:** 10.3389/fpsyg.2024.1476511

**Published:** 2024-10-17

**Authors:** Y. Joel Wong, Alexis L. Pandelios, Kane Carlock, Alexandria M. B. Thielmeyer

**Affiliations:** Department of Applied Psychology in Education and Research Methodology, Indiana University Bloomington, Bloomington, IN, United States

**Keywords:** gratitude, adolescents, social, group, interventions

## Abstract

Most gratitude interventions for adolescents focus on private experiences of gratitude (e.g., gratitude journaling), dyadic expressions of gratitude (e.g., writing a gratitude letter to another person), or group-based psychoeducation about gratitude. By contrast, group interventions that emphasize gratitude social processes (GSPs)—interpersonal or group processes that involve or are directly triggered by the disclosure or expression of gratitude to other group members—provide an ideal forum for adolescents to reap the full benefits of gratitude experiences. In this perspective article, we propose a typology of five GSPs—disclosing, expressing, receiving, responding to, and witnessing gratitude in relation to other group members—that operate synergistically to produce positive effects for adolescents. In turn, we theorize that these GSPs likely produce superior outcomes, as compared to other gratitude interventions, through five psychosocial mechanisms of change: observational learning, group cohesion, vicarious gratitude, group-based gratitude, and collective gratitude. Overall, we encourage researchers and practitioners to incorporate GSPs in their gratitude interventions with adolescents.

## 1 Introduction

How can gratitude interventions for adolescents be improved? In this perspective article, we tackle this question by addressing the vital role of group-based gratitude social processes (GSPs). There has been mounting recent scholarly interest in applying positive psychology to enhance adolescent development. Researchers have emphasized strengths and assets for healthy youth development and created programs to augment adolescents’ well-being (Owens and Waters, 2020; Fernandes et al., 2021; Villacís et al., 2023). Among numerous positive psychology constructs, scholars have increasingly focused on gratitude as a virtue for adolescents to cultivate (Tudge and de Lucca Freitas, 2017). Gratitude is salient to youths because it emerges in early adolescence after children develop the ability to assign meaning to other people’s intentions (Hussong et al., 2019). Indeed, gratitude is relevant to adolescents’ moral, social, and emotional development. Researchers have demonstrated that grateful youth, from diverse communities, have better subjective well-being and prosocial behaviors, as well as lower levels of materialism and antisocial behaviors than their less grateful counterparts (Bono et al., 2019; Chaplin et al., 2019; Kong et al., 2021; Mesurado and Resett, 2024). Additionally, gratitude activities are commonly used in positive psychology interventions in both youth and adult populations (Carr et al., 2021). Gratitude interventions for youth include gratitude journaling, writing gratitude letters to others, and psychoeducation on gratitude (Chaplin et al., 2019; Armenta et al., 2022; Bono et al., 2023). Despite promising findings from some studies (e.g., Bono et al., 2023), a meta-analysis found that, overall, gratitude interventions have produced negligible effects on youth (Renshaw and Olinger Steeves, 2016), a result that dovetails with meta-analytic findings on gratitude interventions in the general population, which includes adults (Davis et al., 2016).

Several explanations account for why gratitude interventions have generally not been effective. Meta-analytic findings demonstrate that self-help interventions, such as gratitude journaling or writing gratitude letters, generally confer weaker effects than face-to-face interventions facilitated by interventionists (Carr et al., 2021). Adolescents who lack willpower or who have interpersonal or cognitive deficits might struggle to complete self-help gratitude activities, whereas a group interventionist can help scaffold participants’ experience in the intervention by providing encouragement, guidance, and feedback (Wong, 2023). Moreover, although most face-to-face gratitude interventions for adolescents involve group activities, these do not typically provide opportunities for participants to express gratitude to other group members (e.g., Duthely et al., 2017).

Against this backdrop, our thesis is that the positive effects of gratitude interventions for adolescents can be strengthened by incorporating group-based GSPs, defined as interpersonal or group processes that involve or are directly triggered by the disclosure or expression of gratitude to others in a group. GSPs are powerful forces of change—instead of simply learning about gratitude (psychoeducation), engaging in private experiences of gratitude (e.g., journaling), or expressing gratitude to individuals outside the group (e.g., writing gratitude letters), the group becomes the fulcrum of gratitude experiences when such social processes are prioritized.

In this article, we propose a typology of five GSPs, theorize salient psychosocial mechanisms through which GSPs generate salubrious outcomes for adolescents (e.g., higher levels of well-being and prosocial behaviors), and review the literature on group interventions that incorporate GSPs for adolescents. Before continuing, we delineate the scope of this article and offer a few clarifications. Although GSPs are helpful to people from all age groups, we focus on youths because adolescence is a formative developmental period in which youths acquire social skills to relate to others appropriately (Merrell and Gimpel, 2014). They are also particularly susceptible to group social norms and peer influence (Busching and Krahé, 2020; Giletta et al., 2021), which shape their prosocial behaviors, such as expressing gratitude to others. Hence, group work offers the ideal forum for youths to experience GSPs and learn gratitude-related social skills. And although some GSPs can occur in interpersonal dyads, we focus on their occurrences in groups, defined as social settings involving three or more people. Following Wong et al. (2024), we adopt a multilevel definition of gratitude, that includes generalized gratitude (appreciating benefits in one’s life and attributing such benefits to external sources), targeted gratitude (attributing one’s benefits to the intentional actions of moral agents), and actionable gratitude (actions that communicate gratitude to one’s benefactors). Although all three levels of gratitude are relevant to group work, actionable gratitude (e.g., orally expressing gratitude to the group) is the most salient of the three levels within group contexts. Regarding the scope of this article, we do not emphasize gratitude cognitions and emotions, given our focus on group-based social processes.

## 2 Typology of gratitude social processes

**Figure 1:**
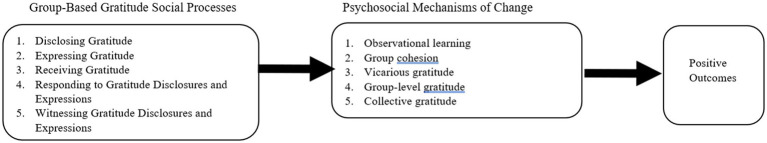
Gratitude social processes and psychosocial mechanisms of change in group interventions.

Here, we propose a typology of five GSPs that can enhance the positive effects of group gratitude interventions: disclosing, expressing, receiving, responding to, and witnessing gratitude in relation to other group members (see Figure 1).

### 2.1 Disclosing gratitude

The first GSP is the disclosure of gratitude, which refers to sharing what one is grateful for with group members (e.g., “I’m grateful our team won the soccer game!”). Research on both gratitude and interpersonal capitalization (the sharing of positive news with others) converge on the notion that disclosing positive or gratitude experiences to others confers several benefits on disclosers (Peters et al., 2018), including higher levels of gratitude (Gray et al., 2024). Lambert et al. (2013) showed that among university students who participated in daily gratitude journaling, those who shared what they wrote with a partner twice a week exhibited greater subjective well-being than those who did not. In another study of parent-adolescent dyads, adolescents’ disclosure of positive experiences to their parents was positively linked to their subsequent high-arousal positive affect (Griffith and Hankin, 2021).

Adolescents’ disclosure of gratitude to others in a group might confer benefits on the disclosers by enabling them to relive, savor, and elaborate on positive experiences linked to gratitude, thereby enhancing its perceived value and accessibility to gratitude-related memories (Lambert et al., 2013; Peters et al., 2018). Moreover, because of its social nature, the disclosure of gratitude offers extra benefits not found in private gratitude journaling. Gratitude disclosures increase the social reality of the discloser’s gratitude experiences; such disclosures can also strengthen the discloser-recipient relationship if the recipient responds positively to the gratitude disclosure as both the recipient and discloser develop a mutual appreciation for each other (Lambert et al., 2013).

### 2.2 Expressing gratitude

The second GSP is the expression of gratitude to other group members. Gratitude expressions differ from gratitude disclosures in that the former involves actionable gratitude; that is, within the group context, an adolescent directly thanks either a group member or the entire group for a benefit they received from them (e.g., “I’m grateful to all of you for supporting me during this difficult time”). A recent meta-analysis demonstrated that gratitude social expression interventions increased psychological well-being for expressers, relative to controls (Kirca et al., 2023). The reasons for such benefits are likely similar to those associated with gratitude disclosures (see 2.1). However, because a sincere gratitude expression tends to have a more positive impact on a recipient than a gratitude disclosure (see 2.3), it confers the added benefit of strengthening the interpersonal bond between the expresser and the recipient (Algoe and Chandler, 2024).

### 2.3 Receiving gratitude

The third GSP is receiving gratitude from another group member. Just as gratitude expressions positively impact the expresser, so do they benefit the recipient of such expressions. Indeed, an adolescent’s sincere gratitude expression to another group member may have a more positive impact on the recipient than a gratitude disclosure, as it demonstrates to the recipient that the expresser values them and they have made a positive difference in the expresser’s life (Aparicio et al., 2022; Wong et al., 2024). Not surprisingly, research has shown that receiving gratitude from another person enhances the recipient’s perceived social impact and self-efficacy (Ni et al., 2022). Interestingly, people who receive gratitude from others also tend to feel more grateful themselves (Walsh et al., 2022), possibly because it fulfills their psychological need for competence and affiliation (Martela and Sheldon, 2019).

### 2.4 Responding to gratitude disclosures and expressions

The fourth GSP—responding to gratitude disclosures and expressions—refers to the social actions people take in direct response to gratitude disclosures or expressions within a group. For example, an adolescent participating in a group intervention might compliment another group member for their gratitude expression to another group member (e.g., “I love the way you thanked Johnny! I could tell you were really grateful to him.”). Alternatively, after another group member’s disclosure of gratitude (e.g., “I’m so thankful I passed my Chemistry test!”), an adolescent might offer a celebratory response (e.g., “Wow! I’m so happy for you! I know how worried you were!’). When people verbally respond to the sharing of good news (including gratitude expressions and disclosures), they experience several salutary outcomes, including more positive emotions, higher self-regard, and increased relationship satisfaction (Conoley et al., 2015; Peters et al., 2018; Reis et al., 2017).

### 2.5 Witnessing gratitude disclosures and expressions

The fifth GSP refers to observing gratitude disclosures and expressions by other group members. Unlike responders, witnesses do not engage in any overt action. When an adolescent discloses or expresses gratitude to the group, all other group members are considered witnesses. While actively responding to gratitude disclosures and expressions can lead to positive results for responders (see 2.4), the mere witnessing of such disclosures and expressions is also sufficient to generate favorable outcomes for witnesses. For instance, witnessing the interpersonal expression of gratitude generates elevation—a positive emotion tied to observing the admirable actions of others—and increased prosocial behaviors in witnesses (Walsh et al., 2022). Within a group, witnesses who feel a strong sense of connection to gratitude disclosers might, through a process of emotional contagion (van Kleef and Fischer, 2016), also experience vicarious gratitude—the emotion of gratitude for the good fortunes of others (Howell et al., 2015).

### 2.6 Positive feedback loop

We posit that, within a group intervention, the five GSPs are powerful forces of change because they operate synergistically to create a positive feedback loop of increased gratitude, well-being, and relational bonds for adolescents. To illustrate, suppose person A expresses gratitude to person B; B might experience gratitude for being thanked and could reciprocate by thanking A. A now perceives B to be responsive to them, thus reinforcing A’s experience of gratitude; both A and B will also likely feel more connected to each other (Algoe and Chandler, 2024). Likewise, research has found that witnesses of this interaction are likely to act more positively toward both the person expressing gratitude and the one receiving it (Algoe and Chandler, 2024). In the following fictitious vignette, we illustrate how a group facilitator might enable the five GSPs to produce synergistic change in a group intervention for adolescents:

Facilitator: *Who would like to share what they are grateful for this week?*

Isabella: *I visited my grandmother in the hospital this week. She’s dying, and I’m sad. But I’m also grateful to her. As I held her hand, I thought about how she has been such a blessing in my life* [Gratitude disclosure: Isabella provides several examples of what her grandmother did for her and begins to cry.].

Facilitator: *I can tell some of you were moved by what Isabella shared. Who would like to tell Isabella what you are feeling?*

Zuri: *I’m grateful to Isabella.*

Facilitator: *Zuri, could you look at Isabella and tell her what makes you grateful to her?*

Zuri [gratitude expression]: *Isabella, thank you for sharing. I’m touched by your gratitude for your grandmother. It made me realize how much I’ve taken my parents for granted. I love them and your story reminded me of all that my parents have done for me, which makes me grateful* [Isabella receives gratitude.]

Facilitator: *Isabella, what is it like to be thanked by Zuri?*

Isabella [gratitude expression]: *Thank you, Zuri! I did not realize anything I said had an impact on others. I really appreciate what you said* [gratitude expression].

[Witnessing gratitude expression: Other group members are moved by this interaction and begin to tear up.]

Facilitator: *What did the rest of you learn from this exchange between Isabella and Zuri?*

Jun [responding to gratitude expression]: *I’m not very good at thanking people. Zuri taught me what to say and how to say it.*

Facilitator [gratitude expression to the group]: *I want to thank all of you. Today, you have been vulnerable with each other. And you have learned to express gratitude to each other. I feel like we have bonded as a group.*

### 2.7 Optimizing the use of gratitude social processes

How can group facilitators optimize the use of GSPs in group interventions to generate a feedback loop of positive outcomes for adolescents? Here, we propose a few guidelines for group facilitators. GSPs are most meaningful when adolescents have the opportunity during group sessions to share their experiences and to get to know other group members. Hence, a purely didactic group program does not optimally facilitate GSPs. Adolescents will have difficulty expressing gratitude to other group members if they do not have opportunities to hear from these members. To this end, we encourage group facilitators to create many opportunities for group members to share their experiences and perspectives with each other. Additionally, because not all adolescents are adept at expressing gratitude to others, group facilitators might have to coach group members on how to express gratitude by offering prompts and examples to help them articulate their grateful feelings (e.g., “Could you elaborate on what she said that made you grateful?”). Moreover, adolescents vary in how they respond to gratitude expressions and disclosures. Not all types of responses will yield beneficial outcomes. Research on interpersonal capitalization has shown that active-constructive responses to good news (i.e., responding with enthusiasm and interest) produce the best outcomes for both the discloser of the good news and the responder (Woods et al., 2015; Peters et al., 2018). Accordingly, a skillful group facilitator could facilitate adaptive responses to gratitude disclosures and expressions by modeling an active-constructive response (e.g., “Hurray! I’m so happy for you!”).

## 3 Theorized psychosocial mechanisms of change

How do GSPs enhance the effectiveness of gratitude interventions for adolescents? Here, we theorize five psychosocial mechanisms of change relatively unique to group processes (see Figure 1). First, GSPs enable adolescents to acquire gratitude-related social skills by observing how the group facilitator and other group members express, disclose, and respond to gratitude (Wong et al., 2017). This process of observational learning may be particularly useful for youths with poor interpersonal skills and who lack adult role models that express sincere interpersonal gratitude. Second, adolescents participating in groups that prioritize GSPs will likely experience higher levels of group cohesion than those in other interventions (Burlingame et al., 2018). When group members spend time getting to know each other through the practice of GSPs, they may develop a group identity and a sense of connectedness not just to individual group members but to the group itself (van Kleef and Fischer, 2016). Third, gratitude group interventions that offer adolescents ample opportunities for GSPs will likely engender more frequent experiences of vicarious gratitude than other gratitude interventions (Howell et al., 2015). As group members develop a shared group identity through the practice of GSPs, they may begin to view the gratitude-related positive events in other group members’ lives as their own, thus multiplying the range of benefits they are grateful for. Finally, the emergence of a group identity and opportunities to witness other group members’ disclosure and expression of gratitude will also trigger experiences of gratitude that differ from how people experience gratitude at the individual level. We anticipate that groups with GSPs will stimulate increased group-based gratitude and collective gratitude in adolescents, relative to other group interventions. Whereas group-level gratitude are gratitude experiences that arise from members’ self-categorization as group members (Smith and Mackie, 2015; e.g., feeling grateful toward the group or feeling grateful for an outcome related to the group), collective gratitude refers to shared experiences of gratitude grounded in the awareness that others within a group are also feeling grateful (Fehr et al., 2017; Goldenberg et al., 2020).

## 4 Research on GSPs and future directions

How often are GSPs utilized in gratitude group interventions to cultivate gratitude in adolescents? In short, very rarely. Here, we review the nascent research in this area. Bono et al. (2023) evaluated a gratitude intervention for high school students, which included a gratitude curriculum and a gratitude app; this app provided opportunities for students and teachers in a class to thank and receive gratitude from each other. Relative to controls, intervention participants reported increased gratitude and subjective well-being over 6 weeks. Nonetheless, because students and teachers were only allowed to send private gratitude messages, GSPs were confined to dyadic exchanges as there were no opportunities for witnesses to observe such gratitude expressions. In another study, Valdez et al. (2022) evaluated a Facebook online group intervention in which high school students could post their gratitude thoughts and letters to the Facebook group and post gratitude expressions to other group members. Compared to controls, intervention participants reported higher levels of motivation and cognitive engagement. In contrast to Valdez’s online group intervention, Gabana et al. (2022) tested a 6-week face-to-face psychoeducational gratitude group program for female high school student athletes. Each session, the group facilitator set aside time for group members to disclose their gratitude to other group members (at the beginning of each session) and to express and receive gratitude in relation to other group members (during the last 5 minutes of each session). Group members reported better mental health, resilience, and coach-athlete relationships at 3-month follow-up. To our knowledge, this is the first gratitude group intervention for adolescents to offer ample opportunities in each group session for members to practice the five GSPs in relation to other group members.

We encourage researchers to build on this emergent scholarship on GSPs in groups for adolescents. Researchers could randomly assign youths to a psychoeducational gratitude group program with opportunities for group-based GSPs vs. the same psychoeducational gratitude group program without group-based GSPs. We anticipate that differences in outcomes between both conditions would be mediated by our five theorized psychosocial mechanisms—observational learning about gratitude expressions, disclosures, and responses, group cohesion, vicarious gratitude, group-based gratitude, and collective gratitude. Furthermore, future research could address individual differences that moderate the impact of GSPs. For instance, given research showing that adolescents’ secure attachment to their parents are positively correlated with their levels of gratitude (Lin, 2020), we hypothesize that secure parental attachment would enhance the effects of GSPs on adolescent outcomes. Researchers could also test the benefits of GSPs in group interventions across diverse cultural groups. Might cultural groups that value direct interpersonal communications benefit more from GSPs than those that prefer indirect communications (Tong and Yuqing, 2020)? Finally, group facilitators likely vary in their ability to facilitate meaningful GSPs; researchers could therefore examine whether group facilitator effects account for meaningful variance in the frequency and quality of GSPs in groups.

## 5 Conclusion

If gratitude is a social virtue (Navarro and Tudge, 2020), it is ironic that GSPs, which emphasize gratitude exchanges among individuals, are rarely practiced in gratitude interventions (Wong, 2023). GSPs are powerful forces of change in group interventions for adolescents because they provide the *in vivo* practice of actionable gratitude within groups. When youths are afforded opportunities to disclose, express, receive, respond to and witness gratitude in relation to other group members, their experiences of gratitude and the positive consequences of such experiences are likely more intense, meaningful, and durable.

## Data Availability

The original contributions are included in this article. Further inquiries may be directed to the corresponding author.
